# State-of-the-art for contrast-enhanced mammography

**DOI:** 10.1093/bjr/tqae017

**Published:** 2024-01-20

**Authors:** Matthew F Covington, Samantha Salmon, Bradley D Weaver, Laurie L Fajardo

**Affiliations:** Department of Radiology and Imaging Sciences, University of Utah, Salt Lake City, UT, 84112, United States; Center for Quantitative Cancer Imaging, Huntsman Cancer Institute, Salt Lake City, UT, 84112, United States; Department of Radiology and Imaging Sciences, University of Utah, Salt Lake City, UT, 84112, United States; Spencer Fox Eccles School of Medicine, University of Utah, Salt Lake City, UT, 84112, United States; Department of Radiology and Imaging Sciences, University of Utah, Salt Lake City, UT, 84112, United States

**Keywords:** contrast-enhanced mammography, CEM, contrast-enhanced spectral mammography, contrast-enhanced digital mammography, CESM, CEDM

## Abstract

Contrast-enhanced mammography (CEM) is an emerging breast imaging technology with promise for breast cancer screening, diagnosis, and procedural guidance. However, best uses of CEM in comparison with other breast imaging modalities such as tomosynthesis, ultrasound, and MRI remain inconclusive in many clinical settings. This review article summarizes recent peer-reviewed literature, emphasizing retrospective reviews, prospective clinical trials, and meta-analyses published from 2020 to 2023. The intent of this article is to supplement prior comprehensive reviews and summarize the current state-of-the-art of CEM.

## Introduction

Contrast-enhanced mammography (CEM) is an emerging technology that is expected to expand screening, diagnostic, and procedural capabilities in breast imaging. For example, as studies have increasingly reported on the limitations of screening mammography for women with dense breast tissue, CEM may prove to be a more accessible and sensitive screening alternative for dense breasts. CEM is additionally being investigated as a method for evaluating extent of disease in newly diagnosed breast cancers, troubleshooting for diagnostic dilemmas on non-contrasted breast imaging, neoadjuvant therapy response assessment, and high-risk screening, including for surveillance following breast conservation therapy.

Contrast-enhanced mammography is not a perfect imaging test. Like other breast imaging techniques such as 2D mammography, tomosynthesis, and molecular breast imaging, CEM exposes individuals to ionizing radiation with theoretical downstream risk of cancer induction. CEM requires the intravenous (IV) injection of iodinated contrast material with potential for contrast reactions including the rare risk of anaphylaxis. Like MRI, CEM may be limited in detection of abnormal enhancement in cases of elevated background parenchymal enhancement. CEM-guided biopsy, while newly clinically available, has yet to see adoption at many centres.

Multiple literature reviews have previously been published on CEM that include discussion of foundational studies that underly current clinical use of CEM.^1–12^ This article will analyse the peer-reviewed literature, emphasizing retrospective reviews, prospective clinical trials, and meta-analyses published from 2020 to 2023. The intent of this article is to supplement prior comprehensive reviews and summarize the current state-of-the-art of CEM, supported by the most recently published data.

## Technical performance of CEM

**Figure 1.**
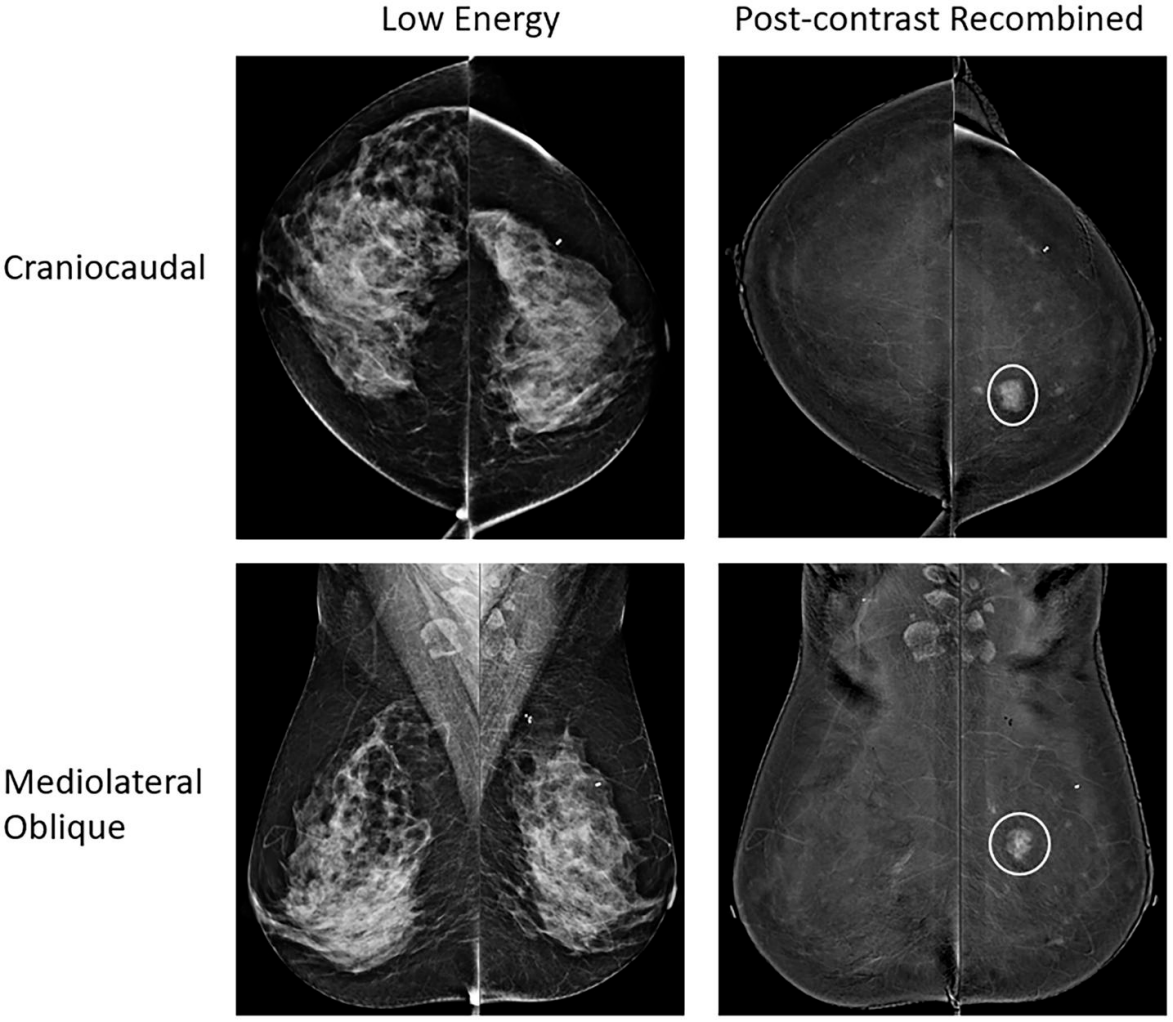
Standard contrast-enhanced mammography views are shown with craniocaudal (top row) and mediolateral oblique (bottom row) views of each breast depicted with low-energy images (right column) and post-contrast recombined images (left column). The white circles show an invasive ductal carcinoma in the medial left breast well-seen on the post-contrast recombined images, not seen on the low-energy images due to masking by dense breast tissue.

A standard CEM exam provides 8 standard images for interpretation, comprised of paired low-energy and post-contrast recombined craniocaudal and mediolateral oblique views of each breast (Figure 1). The low-energy CEM images appear analogous to a 2D mammogram, with a recent retrospective review of 40 cancers reporting potentially improved visualization of these cancers on low-energy CEM images compared with standard 2D mammography.^13^ The post-contrast recombined images are conceptually like a post-contrast subtracted MRI image, albeit obtained in standard mammographic positioning. Each image derives from dual-energy exposures obtained rapidly during a single mammographic compression for each image pair.

Contrast-enhanced mammography imaging acquisition begins approximately 2 min following the intravenous injection of iodinated contrast materials. Imaging is thereafter ideally performed between 2 and 8 min following contrast injection for optimal breast tissue perfusion of contrast material. A prospective study from China evaluating CEM performance for detection of histologically proven malignancy with delayed-phase imaging performed at 7-9 min after injection suggested that the delayed-phase imaging yielded limited diagnostic improvement.^14^ However, whether delayed-phase imaging could be utilized to improve characterization of certain breast cancers with delayed contrast perfusion or washout warrants further investigation.

Many manufacturers also allow tomosynthesis to be added to a CEM exam, although this has not been universally utilized due to increased imaging acquisition times and increased radiation exposure to the breast. When tomosynthesis is added to CEM, the breast receives three exposures per view: the high- and low-energy CEM exposures, and a separate exposure for tomosynthesis. With this approach, the breast positioning and compression are identical for the three acquisitions. Contrast enhancement is not visible on the tomosynthesis images slices.

Many current-generation mammography systems are CEM capable, thereby allowing practices to implement CEM through software and filter upgrades. This negates a need for additional space allocation for CEM equipment other than adequate room for a contrast power injector in the mammography imaging suite.

Interpretation of CEM studies combines a breast radiologists’ mammography and contrast-enhanced breast MRI interpretation skillset into a single examination. Low-energy images appear like a 2D mammogram, and post-contrast recombined images require assessment of background parenchymal enhancement and suspicious enhancing masses, foci, or areas of non-mass enhancement, like breast MRI. A recent publication shows that a standardized lexicon can be implemented with similar agreement to other BI-RADS lexicons currently use for other breast imaging modalities.^15^

More comprehensive descriptions of CEM implementation and technical performance are available in prior reviews.^2–4^^,^^16–20^

## CEM radiation dose

Multiple studies have evaluated radiation exposure from CEM in comparison with 2D mammography and tomosynthesis.^21–24^ For example, Gennaro et al published a dual-centre study demonstrating a 30% increased absorbed dose in CEM compared to standard 2D digital mammography, but a similar dose compared to tomosynthesis.^23^ The authors concluded that CEM use should not be limited based on radiation dose concerns due to the superior diagnostic performance of CEM in comparison with other mammographic techniques.^23^ A separate study comparing radiation exposure from CEM to 2D mammography plus a single tomosynthesis view in 56 patients found a significantly lower average glandular dose for CEM.^24^ Other studies have attempted to delineate radiation dose differences based on breast density. For example, a study evaluating 173 CEM exams reported a lower radiation exposure from CEM in patients with dense breasts compared to individuals with non-dense breasts.^21^ This finding is of significant interest given that CEM is increasingly considered as a potential screening option for individuals with dense breast tissue. CEM techniques that potentially lower radiation dose are under development.^25^ CEM-guided biopsy is also associated with increased radiation exposure though exposure limits remain within Mammography Quality Standards Act Guidelines.^8^ In summary, current radiation exposures from CEM are within acceptable limits for clinical use, and further dose reductions are anticipated.

## CEM-guided biopsy

One of the newest advances in CEM is CEM-guided biopsy capability.^26–30^ CEM has emerged as an alternative method for visualizing breast lesion neovascularity, previously limited to contrast-enhanced breast MRI.^8^ As studies comparing the two methods have shown non-inferiority of CEM to MRI for diagnostic use, a growing number of groups have investigated CEM-guided biopsies as an alternative to MRI-guided biopsies.^26–28^^,^^31^ Possibly the largest study to date by Alcantara et al, reports a high a procedure success rate of 95% and a lower lesion non-visualization rate at time of procedure compared to reported values of MRI-guided breast biopsies.^26^ With this early success, CEM-guided biopsies will likely expand in tandem with growth of CEM for screening and diagnostic use.

The greatest advantage of CEM-guided biopsies arises from the potential increased accessibility for patients.^30^ In a study of 153 enhancing lesions on CEM in 144 patients, only 47 (31%) had a correlate on targeted ultrasound^32^ supporting the need for direct CEM-guided biopsy. Availability of CEM biopsies, however, is limited insofar that FDA-approval was only recently obtained for clinical use in 2020 and many centres with CEM may not yet have CEM-biopsy capability.^27^

An advantage of CEM-guided biopsies is a shorter average procedure time, reported at 15 min, nearly one-third shorter than reported procedure times under MRI guidance.^26^ Improved patient comfort and preference for diagnostic CEM compared to MRI is expected to be similar in the procedural setting as that reported for diagnostic imaging applications.^33^^,^^34^

Additional limitations for CEM-guided biopsies parallel those of diagnostic CEM, namely the risks associated with iodine-based contrast reactions and contrast-induced renal insufficiency, although absolute risk of reaction is low.^8^^,^^35^

## Evaluation of the symptomatic breast

Several recent studies have evaluated accuracy of CEM for evaluation of clinical breast complaints such as focal breast pain or a palpable lump.^36–38^ For example, a review of CEM compared to targeted ultrasound for 115 symptomatic patients with dense breasts showed comparable diagnostic performance of CEM and targeted ultrasound.^36^ These results are concordant with a prior retrospective study evaluating 147 palpable breast abnormalities in 138 patients in which both ultrasound and CEM accurately detected each of the 38 biopsy-proven malignancies.^37^ Of the 13 palpable masses visible on ultrasound but negative on CEM, each mass was found to be benign in nature.

A prospective study comparing CEM to mammographic and ultrasound evaluation of 166 breast lesions in 130 symptomatic patients showed improved sensitivity for cancer detection in dense breasts with CEM, with sensitivity of 97%, compared to a sensitivity of 76% for 2D synthetic mammography. The sensitivity was additionally higher than the reported 83% sensitivity for tomosynthesis and 89% sensitivity for tomosynthesis plus ultrasound.^38^ These studies suggest that CEM could potentially act as stand-alone imaging for evaluation of palpable masses. Further prospective controlled trials are needed to further elucidate these findings in larger patient populations.

## Locoregional pre-surgical staging of breast cancer

Multiple recent studies have assessed the diagnostic capabilities of CEM for pre-surgical staging of newly diagnosed breast cancers.^39–46^ A 2022 study comparing CEM and 2D digital mammography reported that CEM detected an additional 19 lesions in 13 patients with 9/19 (47%) yielding malignant results.^39^ A separate study reported that CEM for pre-operative staging caused additional breast imaging in 23% of patients, yielding additional biopsies in 18% of patients with 49% of biopsies demonstrating malignancy.^40^ This additional CEM imaging altered surgical planning in up to 18% of patients (9% converting from breast conservation therapy to mastectomies) with no conversions to mastectomies resulting from false-positive CEM findings.^40^ The most common reason for CEM not identifying an index cancer on pre-operative imaging was location of a lesion outside of the CEM field-of-view.^40^

A separate study of 231 patients showed a similar change in surgical plan in 33/231 patients (25%) based on additional CEM evaluation.^44^ In a multi-centre study from Singapore and Taiwan of 200 women (96% with dense breasts) with 232 sites of malignancy, CEM altered surgical plan in 36 patients (18%) compared to mammographic and sonographic evaluation, with false-positive findings identified in 13/200 patients (7%).^43^ A multi-reader study on pre-operative staging of 78 patients with 100 lesions comparing CEM to diagnostic mammography plus tomosynthesis showed a higher detection rate of additional sites of malignancy on CEM, most notably in dense breasts.^42^ A Polish study of 60 biopsy-proven breast cancers demonstrated that both CEM and MRI detected areas of multifocality and multicentricity at a similar rate, resulting in altered surgical planning in 25% of cases compared to evaluation with non-contrasted modalities.^45^

A prospective study from Australia compared CEM to MRI for breast cancer staging in 59 women with 68 sites of malignancy and demonstrated statistically equivalent sensitivities of 99% and 97%, respectively.^46^ This study showed a sensitivity of 66/68 lesions (97%) for MRI and 67/68 (99%) for CEM for the index lesion.^46^ However, when considering 41 additional lesions detected in 29 patients by either MRI or CEM, with 6/41 proved by biopsy to be malignant, CEM detected only 1/6 additional malignancies compared to 6/6 for MRI.^46^ This suggests that CEM may not be as sensitive as MRI for detection of additional occult lesions on standard of care mammogram and ultrasound.

**Figure 2.**
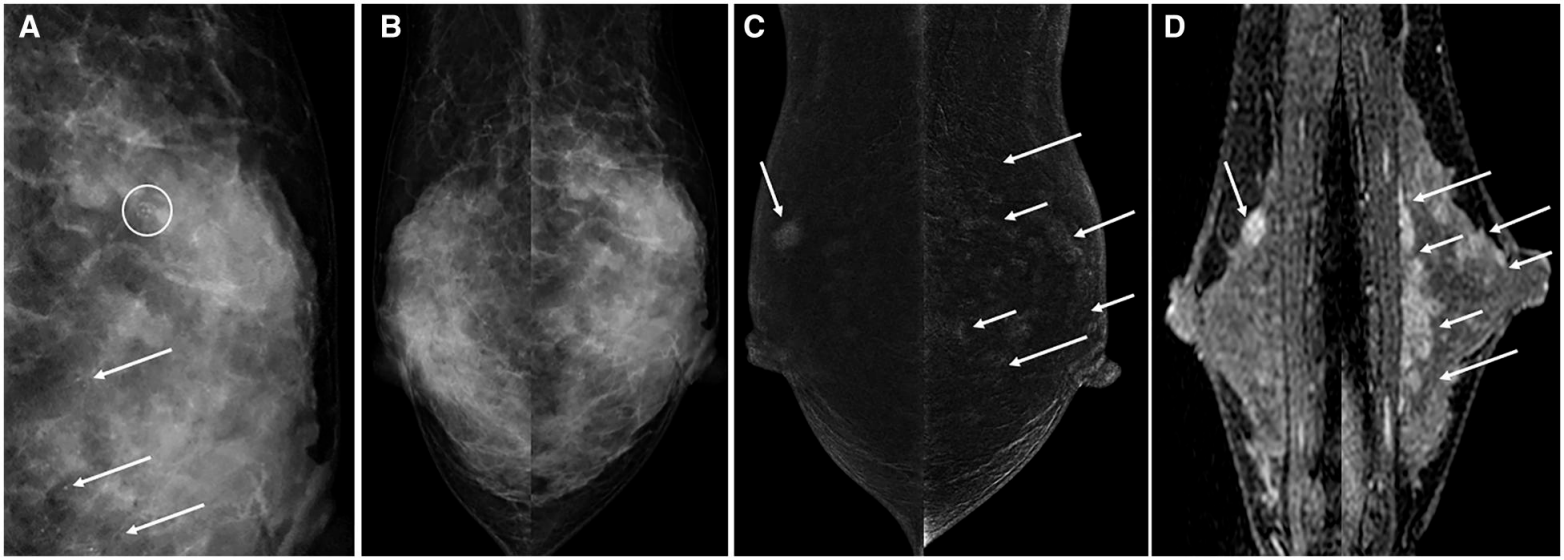
33-year-old woman with left breast calcifications found to represent ductal carcinoma in situ (DCIS) following core needle biopsy. Both CEM and MRI were performed for pre-surgical locoregional staging. (A) Left breast lateral magnification view demonstrates grouped mildly pleomorphic calcifications (white circle) and other more scattered round calcifications in the breast. (B) Low-energy CEM images in mediolateral oblique (MLO) projection show extremely dense breast tissue in both breasts with no additional significant mammographic finding. (C) Post-contrast recombined CEM images demonstrate widespread non-mass enhancement throughout the left breast on the MLO view suggesting multicentric DCIS (white arrows). An oval mass is also seen on the right MLO view which was not seen on standard mammography. (D) T1-fat saturated post-contrast sagittal breast MRI views demonstrate a similar degree of multicentric non-mass enhancement in the left breast (white arrows), and an oval enhancing mass in the right breast (white arrow). Final surgical pathology demonstrated 5.4 cm of multicentric DCIS in the left breast, corresponding with the extent of non-mass enhancement on CEM and MRI, and a right breast fibroadenoma accounting for the additional enhancing oval mass in the right breast on CEM and MRI.

These studies suggest the use of CEM in pre-surgical planning will offer similar benefit and rate of change to the surgical plan compared to MRI and improved evaluation of extent of disease compared to 2D mammography, tomosynthesis, and ultrasound, though MRI may remain more sensitive than CEM for detection of additional foci of malignancy occult on standard mammography and ultrasound. On the other hand, a United Kingdom study demonstrated cost-savings to the health care system when switching from breast MRI to CEM for pre-surgical staging.^47^ An example CEM study for locoregional pre-surgical staging is shown in Figure 2.

## Evaluation of indeterminate findings on other diagnostic breast imaging studies

Contrast-enhanced mammography is additionally being evaluated as a tool to further characterize breast findings that would otherwise be considered indeterminate.^48–51^ Several groups have investigated whether CEM can further characterize calcifications indeterminate on diagnostic mammography as benign or malignant. Amir et al observed that calcifications with associated enhancement are associated with higher malignancy rates; however, there is limited negative predictive value.^48^ A separate study of 74 calcified lesions showed that the addition of the low-energy image with calcifications lowered specificity and that contrast-enhanced images, as well as machine learning, may improve characterization of BI-RADS 4 calcified lesions compared to low-energy images alone.^51^ Depretto et al, however, reported a low sensitivity of 46% in a study of 36 clusters of calcifications with abnormal enhancement at 7 of 15 sites (46%) of biopsy-malignant calcifications.^52^ Of sites of malignancy not enhancing on CEM, 75% had lesions less than 5 mm with Ki-67 values under 5% suggesting lesions below a certain size threshold may not benefit from CEM characterization.^52^ Further studies assessing the best uses and limitations of CEM to characterize indeterminate breast calcifications as benign or malignant would be helpful.

For individuals with dense breast tissue, CEM has been proposed as a tool to evaluate lesions indeterminate on mammography and ultrasound.^49^ An Egyptian study assessed 171 BI-RADS 3 or 4 lesions demonstrating slightly higher sensitivity of MRI compared to CEM (100% and 94% respectively, *P* = .014) but statistically equivalent accuracy (91% MRI and 85% CEM, *P* = .134), with the authors concluding CEM is a viable alternative to MRI.^50^ These results were reproduced in a similar study from Yüzkan et al.^53^

A study evaluating use of CEM as a second look modality to identify correlates for suspicious or indeterminate MRI findings showed that CEM detected a higher fraction of MRI lesions in comparison with ultrasound with 76/109 (70%) lesions identified on CEM and 50/109 (46%) lesions on identified on ultrasound (*P* < .001).^54^ This suggests that CEM could be an alternative or complementary method to ultrasound or mammography for sampling MRI-detected lesions.

## Diagnostic evaluation of screening recalls

Several recent studies have evaluated performance of CEM to further characterize findings recalled from screening mammography.^55^^,^^56^ An Italian study of 207 patients recalled from screening mammography and evaluated with CEM reported a 94% sensitivity and a 66% specificity for malignancy detection, with a reduced biopsy rate of 16% compared to standard-of-care evaluation.^56^ A separate study from Egypt suggests that CEM may provide particular value in evaluating mammographic asymmetries.^55^ Necosia et al suggest CEM has improved specificity for benign cysts compared to traditional mammography via internal non-enhancement, potentially lowering screening recall rates.^57^ Thus, CEM for diagnostic evaluation of screening recalls may increase both sensitivity and specificity, and lower biopsy rates, compared to standard of care evaluation. However, whether CEM is superior to MRI for this indication is currently unclear. A single-institution study comparing CEM with MRI for lesions rated BI-RADS 3-5 on standard-of-care evaluation with mammography and/or ultrasound showed the best results in diagnostic accuracy for benign and malignant breast lesions were obtained with dynamic contrast-enhanced breast MRI (sensitivity 82% and specificity 80% for CEM; sensitivity 96% and specificity 88% for MRI).^58^

## Neoadjuvant therapy response assessment

**Figure 3.**
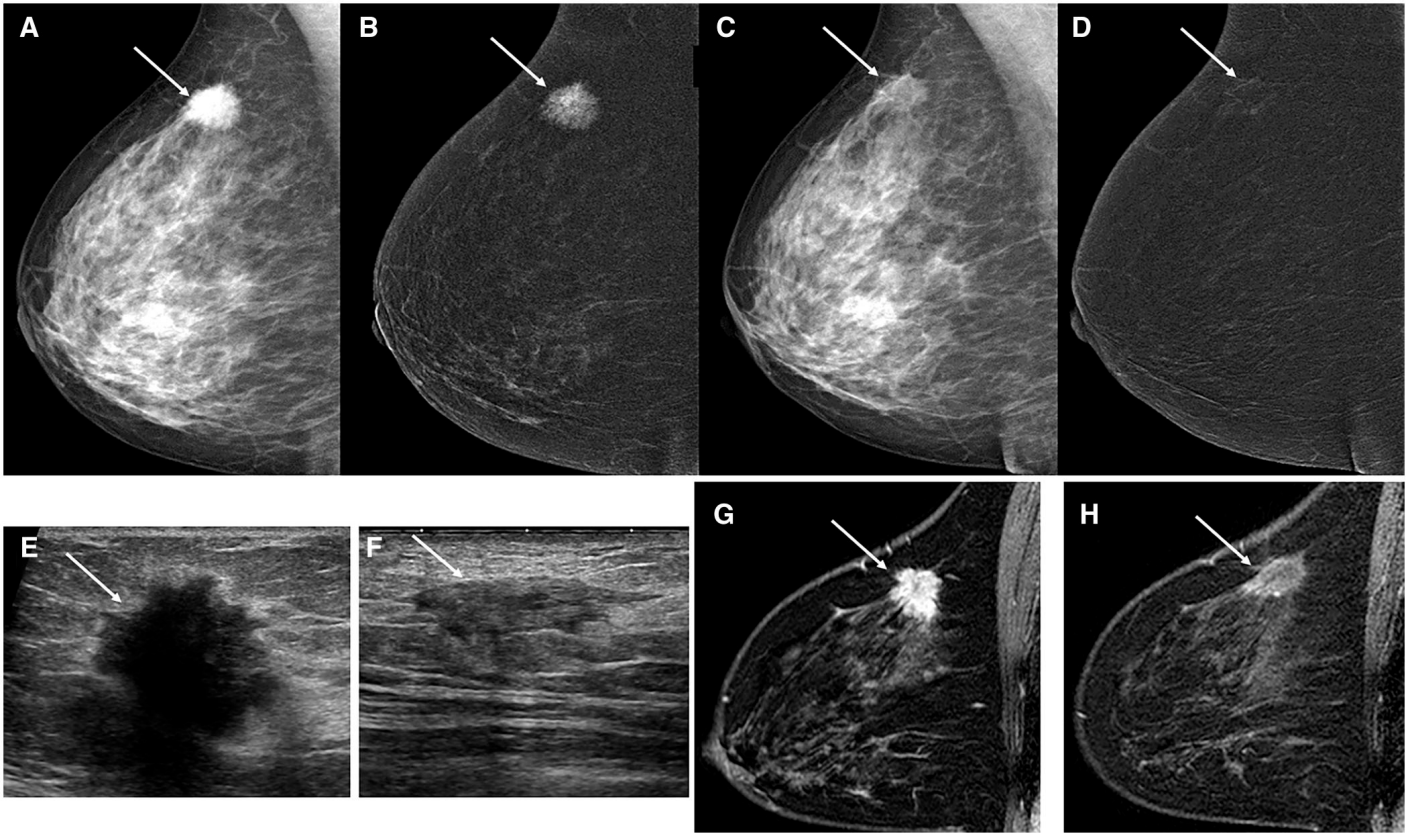
53-year-old woman with histologically proven right breast invasive ductal carcinoma undergoing diagnostic imaging evaluation for response to neoadjuvant therapy. (A) Low-energy right breast mediolateral oblique (MLO) CEM image demonstrates a hyperdense spiculated mass in the superior posterior breast (white arrow) compatible with histologically proven right breast invasive ductal carcinoma. (B) Post-contrast recombined MLO view of the right breast shows the spiculated mass intensely enhances (white arrow) compatible with malignancy. (C) Low-energy MLO view of the right breast following neoadjuvant therapy demonstrates reduced size and density of known malignancy (white arrow) compatible with response to therapy. (D) Post-contrast recombined MLO view of the right breast following neoadjuvant therapy demonstrates mild residual enhancement at site of known malignancy (white arrow) suggesting treatment response with residual viable malignancy. (E) Right breast ultrasound image prior to neoadjuvant therapy demonstrates an irregular hypoechoic mass with posterior acoustic shadowing compatible with known malignancy. (F) Right breast ultrasound image following neoadjuvant therapy demonstrates reduced size of the known malignancy with some residual mass-like components (white arrow) suggesting partial treatment response. (G) T1-subtracted post-contrast MRI sagittal view of the right breast prior to neoadjuvant therapy demonstrates an intensely enhancing mass (white arrow) compatible with known malignancy. (H) T1-subtracted post-contrast MRI sagittal view of the right breast following neoadjuvant therapy demonstrates mild residual enhancement at site of known malignancy suggesting treatment response with residual viable malignancy. Final surgical pathology demonstrated treatment response of known invasive carcinoma with residual viable tumour compatible with the imaging findings on CEM, ultrasound, and MRI of partial response to therapy.

The ability of CEM to evaluate response of known breast cancer to neoadjuvant therapy (example case in Figure 3) has been assessed in several recent studies.^59–62^ A Chilean study concluded that CEM compares favourably to MRI for response to neoadjuvant therapy for locally advanced breast cancer.^59^ A separate study of 110 participants with 115 breast cancers showed CEM compared favourably to MRI for assessment of residual disease on pathology.^60^ A study evaluating CEM assessment of residual disease following neoadjuvant therapy specifically for calcified lesions showed that CEM yielded a sensitivity of 96% for residual disease but demonstrated a low specificity of 14.3% when CEM was used in addition to standard mammography due to the inclusion of calcifications on the low-energy CEM images.^61^ A retrospective Polish study evaluating 63 patients concluded low-energy CEM images may overestimate size of residual malignancy by an average of 6 mm, whereas post-contrast recombined CEM images may underestimate lesion size by approximately 3 mm.^62^ A survey of 18 patients who underwent contrast-enhanced digital breast tomosynthesis and breast MRI showed 77% preferred contrast-enhanced digital breast tomosynthesis.^33^ These studies show potential utility of CEM for neoadjuvant therapy response assessment, though CEM may have low specificity for calcified lesions, and may slightly over- or under-estimate size of residual malignancy according to whether low-energy or recombined images are used for measurement, respectively.

## CEM as an alternative to contrast-enhanced breast MRI: data from meta-analyses

Recent meta-analyses evaluating the viability of CEM as an alternative to MRI generally report a similar sensitivity, specificity, and accuracy between these examinations.^63^^,^^64^ A separate meta-analysis of 7 studies with 1137 lesions had concordant results concluding contrast-enhanced breast MRI had a higher sensitivity for breast cancer detection than CEM, 97% and 91%, respectively (*P *<* *.001), with statistically equivalent specificity, 69% and 74%, respectively (*P* = .09).^35^ Given that the sensitivity of CEM remains higher than non-contrasted modalities and is near that of MRI in early data, CEM will continue to be investigated and used as an alternative to contrast-enhanced breast MRI.

## Breast cancer screening including supplemental screening of dense breasts

There is interest in the use of CEM as a screening modality for individuals with dense breast tissue with intermediate lifetime risk of breast cancer.^16^^,^^24^^,^^63^^,^^65^ A recent meta-analysis of 15 studies comparing CEM and contrast-enhanced breast MRI concluded that both have increased sensitivity for breast malignancy compared to standard imaging (97% sensitivity for CEM and 96% sensitivity for MRI), concluding CEM is a viable alternative to breast MRI for supplemental screening in dense breasts.^63^ Using CEM for individuals at intermediate risk due to history of lobular neoplasia yielded high accuracy, sensitivity, and specificity in a recent review of 132 women with 306 CEM examinations.^66^ A Polish study reported CEM and MRI performed with similar accuracy in lesion detection suggesting CEM could be used as an alternative to MRI for individuals with dense breasts.^67^ Availability of CEM-guided biopsy has been reported as potentially helpful to support the expanded use of CEM for dense breast screening.^30^

The Contrast Enhanced Mammography Imaging Screening Trial (CMIST) (NCT 05625659) is a multi-centre prospective trial on use of CEM for dense breast screening with anticipated enrolment of 2032 patients that is currently underway.^11^

Of note, breast cancer screening with CEM has been recommended by the American College of Radiology as a potential alternative for individuals at high lifetime risk of breast cancer who cannot undergo MRI.^65^^,^^68^

## Surveillance of breast cancer following definitive surgical treatment

A few recent studies have evaluated use of CEM for surveillance of breast cancer following definitive surgical management as a means of detecting recurrence and to better evaluate areas of scar tissue at a surgical site.^69^^,^^70^ In a study of 1191 patients receiving their first surveillance CEM, 73 patients were recalled yielding 38 cancer diagnoses (28 invasive, 9 DCIS), of which 41% were detected only on the post-contrast recombined image.^69^ A separate study comparing imaging surveillance with CEM to 2D mammography demonstrated an increased cancer detection rate of 15.4 per 1000 exams compared to 6.2 per 1000 exams on 2D mammography.^70^ These studies suggest potential clinical benefit of CEM for detection of breast cancer recurrence following definitive surgery.

## CEM for individuals with breast implants

Contrast-enhanced mammography has been evaluated for pre-operative staging in individuals with breast augmentation as a potential alternative to breast MRI.^71^^,^^72^ In a study of 17 women with newly diagnosed breast cancer with implant breast augmentation, CEM detected all six additional malignancies that were identified on breast MRI.^71^ A separate study on 104 women with breast implants who underwent 198 CEM exams for various indications concluded that it is feasible to use CEM for diagnostic and screening purposes in individuals with breast implants.^72^

## Background parenchymal enhancement

Like contrast-enhanced breast MRI, CEM may show benign background parenchymal enhancement of varying intensity on post-contrast recombined CEM images. A recent study reports that CEM background parenchymal enhancement was negatively associated with age, prior history of breast cancer, breast cancer treatment, and post-menopausal status and positively associated with increasing breast density, pre-menopausal status, and irregular menstrual cycles.^73^ CEM background enhancement is reported to be higher in pre-menopausal individuals with dense breast tissue.^74^ Background parenchymal enhancement is the most suppressed during the oestrogen surge of the follicular phase (days 8-14) of the menstrual cycle, per a retrospective review of CEM studies on 207 patients.^73^ A study in 69 patients with known breast cancer evaluated with CEM and MRI showed that the accuracy of CEM was significantly superior to MRI in women with minimal to mild background parenchymal enhancement (sensitivity of 91% and specificity of 92% for CEM vs sensitivity of 90% and specificity of 71% for MRI, *P* = .002).^75^

Quantitative measurements of lesion-to-background enhancement have been reported to help in differentiating benign and malignant lesions.^76^ Several scoring systems have also been proposed based on CEM enhancement to improve characterization of CEM-detected lesions as benign or malignant.^57^^,^^77^^,^^78^ A potentially improved CEM reconstruction algorithm that may reduce or eliminate artefacts on CEM images while preserving true contrast enhancement is also under study.^79^

## Artificial intelligence

Multiple recent studies have assessed the ability of artificial intelligence (AI) to aid CEM interpretation.^80–97^ Specifically, these studies report that radiomics and other AI methods may help differentiate areas of breast malignancy from background parenchymal enhancement or other benign entities,^81–90^ aid in neoadjuvant therapy response assessment,^91^^,^^92^ identify breast cancer invasion, hormone receptor status, and tumour grade,^93^^,^^94^ identify HER2-positive and triple-negative breast cancers,^95^ and predict presence of axillary lymph node metastases.^96^ Several of these studies show high accuracy of AI for differentiating benign from malignant breast lesions. For example, an attention-based deep learning model that evaluated 1,239 CEM exams with 805 malignant lesions and 288 benign lesions in a multi-centre study reported a sensitivity of 85% and a specificity of 100% for characterizing benign from malignant breast lesions.^97^

## Conclusion and further directions

The state-of-the-art for CEM includes potentially expanded use for emerging indications, including use for breast cancer screening in dense breasts, and evaluation of findings recalled for additional evaluation from screening mammography (Table 1). More established uses of CEM are further supported by recent literature such as CEM use for pre-operative breast cancer staging, neoadjuvant therapy response evaluation, and for troubleshooting findings indeterminate on other diagnostic breast imaging studies. Additional investigations on use of CEM-guided biopsy will inform and likely support an expanded use of CEM for breast cancer screening, diagnosis, and biopsy. Multiple recent investigations suggest AI and radiomics may aid human interpretation of CEM images, and we anticipate these techniques will be adopted for clinical use pending further advancements and continued research.

**Table 1.** Established and emerging indications for contrast-enhanced mammography (CEM).

Established CEM indications	Locoregional pre-surgical staging of breast cancer Neoadjuvant therapy response assessment Evaluation of indeterminate findings on other diagnostic breast imaging studies Evaluation of symptomatic breast Surrogate to breast MRI when breast MRI is contraindicated
Emerging CEM indications	CEM-guided biopsy Breast cancer screening including intermediate-risk dense breast and high-risk screening settings Surveillance of breast cancer following definitive surgical management Artificial intelligence and radiomics to aid CEM interpretation Diagnostic evaluation of screening recalls CEM-as alternative to contrast-enhanced breast MRI outside of MRI contraindication

Much of the current data on CEM accuracy stems from retrospective studies in populations of individuals with histologically proven breast cancer. Prospective data evaluating CEM performance for known breast cancer evaluation, evaluation of the symptomatic breast, and screening dense breasts of various lifetime risk thresholds are necessary. For example, a recent meta-analysis of CEM literature only including prospective trials showed an overall sensitivity of 85% and a specificity of 77% for detection of breast cancer in women with clinical or radiological suspicion.^98^ The authors state that these results show a slightly lower sensitivity and higher specificity compared to previously available data, potentially due to evaluating prospective studies only and excluding studies performed solely on patients with known breast cancer.^98^ Likewise, the meta-analysis by Potsch et al^35^ mentioned earlier concluded that MRI had a higher sensitivity for breast cancer detection than CEM. This questions what may be a common belief that CEM sensitivity is equivalent to breast MRI. For comparison, a separate meta-analysis including both prospective and retrospective studies comparing the performance of CEM and MRI for breast cancer diagnosis showed a slight superiority for CEM.^99^ It is also worth considering that recent studies suggest that malignancies less likely to enhance on CEM include many that are calcified, and especially those that are smaller than 5 mm in size with a low Ki-67 value.^52^ Thus, cancers missed on CEM may be small and biologically indolent cancers that are less likely to harm patients.

Current barriers to clinical implementation include risks of iodinated contrast media, increased radiation exposure, workflow considerations, reimbursement challenges, and a need for additional evidence to support use for various clinical indications.^100^ Acknowledging these barriers, CEM remains a breast imaging study with great potential for a wide variety of applications for breast cancer screening, diagnosis, and biopsy. Despite this promise, CEM remains unavailable at many breast imaging centers, perhaps due to a lack of guidelines to inform best clinical uses, limited by mostly retrospective data. Wider adoption of CEM will likely be influenced by ongoing prospective, multi-centre clinical trials, such as CMIST.
